# GEDI: a user-friendly toolbox for analysis of large-scale gene expression data

**DOI:** 10.1186/1471-2105-8-457

**Published:** 2007-11-19

**Authors:** André Fujita, João R Sato, Carlos E Ferreira, Mari C Sogayar

**Affiliations:** 1 Chemistry Institute, University of São Paulo, Av. Lineu Prestes, 748 – São Paulo, 05508-900, SP, Brazil; 2 Institute of Mathematics and Statistics, University of São Paulo, Rua do Matão, 1010 – São Paulo, 05508-090, SP, Brazil

## Abstract

**Background:**

Several mathematical and statistical methods have been proposed in the last few years to analyze microarray data. Most of those methods involve complicated formulas, and software implementations that require advanced computer programming skills. Researchers from other areas may experience difficulties when they attempting to use those methods in their research. Here we present an user-friendly toolbox which allows large-scale gene expression analysis to be carried out by biomedical researchers with limited programming skills.

**Results:**

Here, we introduce an user-friendly toolbox called GEDI (Gene Expression Data Interpreter), an extensible, open-source, and freely-available tool that we believe will be useful to a wide range of laboratories, and to researchers with no background in Mathematics and Computer Science, allowing them to analyze their own data by applying both classical and advanced approaches developed and recently published by Fujita et al.

**Conclusion:**

GEDI is an integrated user-friendly viewer that combines the state of the art SVR, DVAR and SVAR algorithms, previously developed by us. It facilitates the application of SVR, DVAR and SVAR, further than the mathematical formulas present in the corresponding publications, and allows one to better understand the results by means of available visualizations. Both running the statistical methods and visualizing the results are carried out within the graphical user interface, rendering these algorithms accessible to the broad community of researchers in Molecular Biology.

## Background

High-throughput DNA microarray technologies yield up to tens of thousands of gene expression data, which are useful to identify differentially expressed genes, biomarkers and molecular disease profiles. In recent years, microarray platforms have become available at relatively low costs, becoming more popular among research groups which are interested in gene expression analysis. On the other hand, much effort has been spent in developing improved methods to analyze the data derived from these microarrays. These methods involve advanced mathematical and statistical models, which are quite cumbersome to biomedical researchers who attempt to implement these methods. Due to this difficulty, some of these advanced methods are often abandoned and data analysis is carried out using only the classical methods, which are implemented in popular statistical softwares. An user-friendly software could make it possible to use recently developed methods to integrate, qualify, and infer biological insights from gene expression data.

Our aim is to provide a toolbox named GEDI containing an user-friendly interface and advanced statistical methods to analyze data derived from DNA microarrays.

We have previously developed the following analytical approaches:

1. SVR (Support Vector Regression) [1] – a microarray data normalization method published in this journal, is based on a non-parametric regression, namely, Support Vector Regression, which is more robust to outliers (differentially expressed genes), therefore, this approach is superior to classical methods, such as Loess, to identify differentially expressed genes even for very lowly or very highly expressed genes, for which the expression variance is high.

2. DVAR (Dynamic Vector Auto Regressive Model) [2] – a gene expression regulatory network inference model based on time-series data, avoiding stationarity and linearity assumptions, since it is well known that different cell cycle phases involve different circuits. Hence, using DVAR, it is possible to infer different connectivities occuring during different cell cycle phases. The DVAR approach does not require model pre-specification being, therefore, unbiased. The inferred connectivities are causalities based on the Granger causality concept. This is naturally applied to networks containing cycles (feedback mechanisms).

3. SVAR (Sparse Vector Auto Regressive Model) [3] – similarly to DVAR, SVAR is also a gene expression regulatory network inference method based on time-series data. It is an extension of the VAR (Vector Autoregressive) method, consequently, it infers Granger causalities with the advantage that it is possible to infer and statistically test the connectivities under the following context: when the number of samples (microarrays) is lower than the number of parameters (genes), which is a very frequent condition nowadays.

SVR, DVAR and SVAR were available until now only as mathematical formulas, as described in the corresponding publications. Here, we introduce GEDI (Gene Expression Data Interpreter) Version 1.0 as an integrated software, providing easy access to the SVR, DVAR and SVAR algorithms, as well as to some other tools for gene expression data analysis. GEDI has an user-friendly interface and visualization capabilities to facilitate data interpretation.

## Implementation

The current version (GEDI 1.0) (see Additional file 1) runs on Windows and Linux operating systems and requires pre-installation of the R environment and of some R packages, which are freely available for downloading at [4]. GEDI was entirely implemented in the R statistical language, being available upon GPL license. Source code, installation instructions, tutorials and some example input datasets are available at the GEDI's website. Since GEDI is an open source software, new tools can easily be added, allowing researchers the flexibility of implementing new functionalities, according to their own needs.

## Results and discussion

The GEDI toolbox provides an user-friendly environment to perform both well-known basic analysis and advanced methods published in the last few years. GEDI allows the analysis of gene expression data in four major steps, starting from eliminating the bias generated by the microarray technique (normalization step), followed by identification of differentially expressed genes, classification of samples based on molecular profiles to identify potential biomarkers or targets for drugs, and, finally, inferring gene functionality by constructing gene expression regulatory networks.

1. **Microarray data normalization: **global and quantile [5] normalization methods are implemented in this version of GEDI. In addition, several normalization methods based on non-parametric regressions are also implemented, comprising the following methods: Loess [6], Splines [7,8], Wavelets [9] and SVR (Support Vector Regression) [1]. Also, for more than two microarrays, the cyclic normalization is performed as described in [5].

2. **Identification of differentially expressed genes: ***t*-test, *t*-test with permutation and the non-parametric Wilcoxon test with FDR (False Discovery Rate) [10] adjustment are available. Moreover, the recently published SAM (Significance Analysis of Microarray) is also available [11]. Putative differentially expressed genes are listed from the lowest to the highest significant FDR-adjusted p-value.

3. **Samples clustering and classification: **often, a clinical interest requires identification of biomarkers, which may discriminate between pathological and normal samples. Therefore, GEDI has implemented the *k*-means clustering method, linear/quadratic Fisher discriminant analysis [12], hierarchical clustering [13] and the recently described SVM (Support Vector Machine) approach [14] with a cross-validation procedure.

4. **Construction of gene regulatory networks: **usually, it is of interest to identify which pathways the identified genes are related to. Unfortunately, depending on the treatment conditions, cell lines or tissues, these pathways have not yet been studied or are not yet known. GEDI offers some approaches to infer regulatory networks, based on gene expression data, with no *a priori *additional biological information. The methods employed are Pearson and Spearman partial correlation analysis to infer instantaneous associations and advanced methods based on Granger causality [15], such as VAR (Vector Autoregressive) [16], DVAR (Dynamic Vector Autoregressive) [2] and SVAR (Sparse Vector Autoregressive) [3]. The VAR methods are of great interest because they allow infering Granger causalities from time-series gene expression data. DVAR may infer time (cell cycle)-varying connectivities, while SVAR may allow constructing large networks from only a few samples.

**Figure 1:**
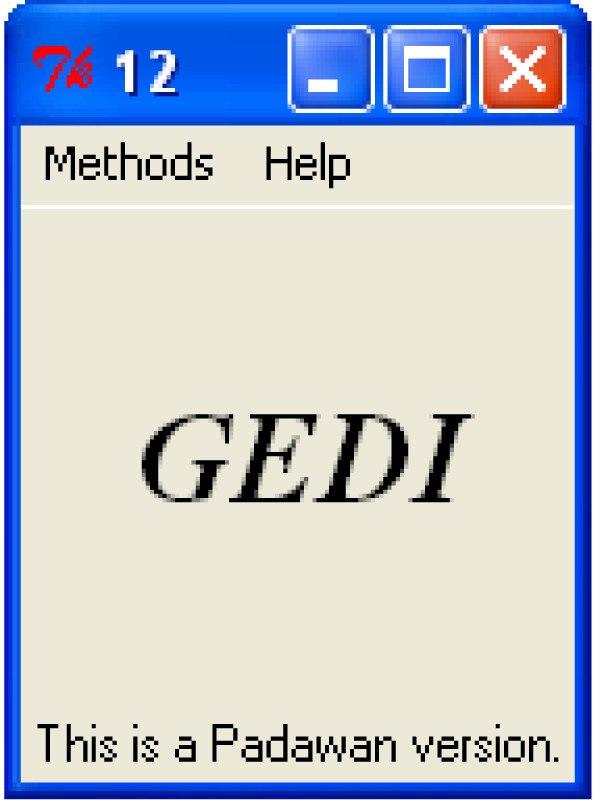
**Snapshot of the GEDI interface**. The GEDI interface is very simple and practical. One may select the desired analysis tool and then, an interactive window will open requesting the desired parameters for the selected method.

Figure 1 illustrates the GEDI interface. The user-friendly interface allows that, with a few clicks, the user may access any analitical method implemented in GEDI. The graphical user interface (GUI) is displayed using the Tcl/Tk library, opening interactive windows where it is possible to easily input the parameters required for each method.

The input data format is very simple and independent of the microarray platform, *i.e*., it should consist of text files organized in a matrix, where each column is one microarray and each row is represented by one gene. To facilitate for the user, the input files have the same format for all functionalities.

The outputs are composed by graphics and numerical results. The plots may be saved as vectorial postscript files, allowing zoom without losing resolution. The numerical results may be saved in a plain tab delimited text file, which may be viewed using any text editor.

**Figure 2:**
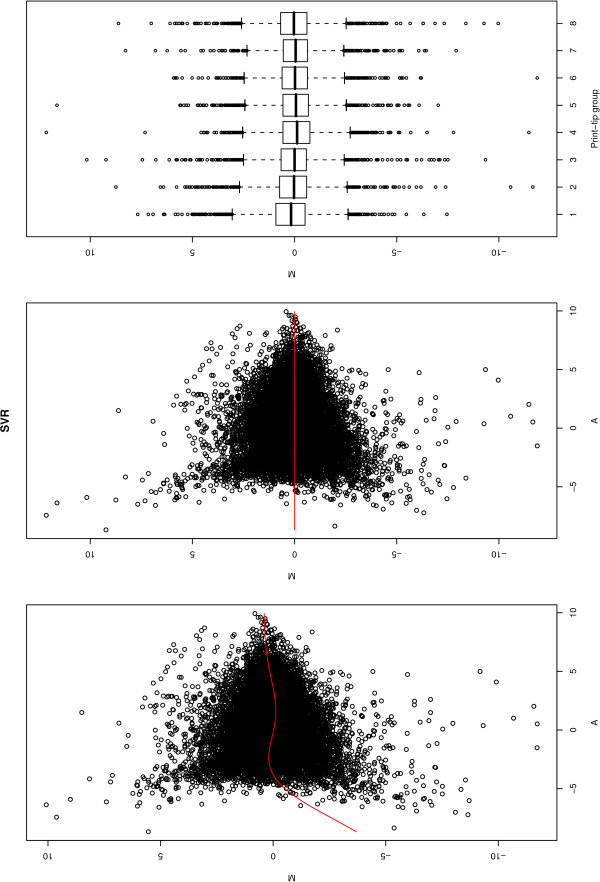
**Microarray normalization**. A MA plot calculated using the SVR normalization method. From left to right are illustrated the raw data, normalized data and the print-tips.

**Figure 3:**
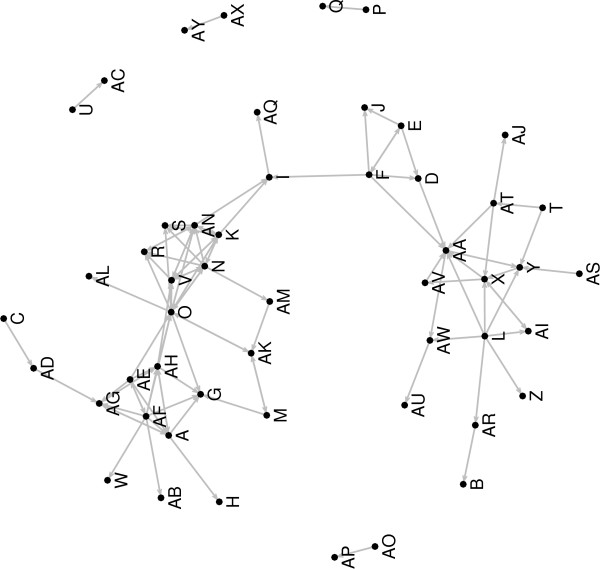
**SVAR output**. The gene expression regulatory network generated by SVAR, where each node is a gene and the oriented edges represent the Granger causalities.

**Figure 4:**
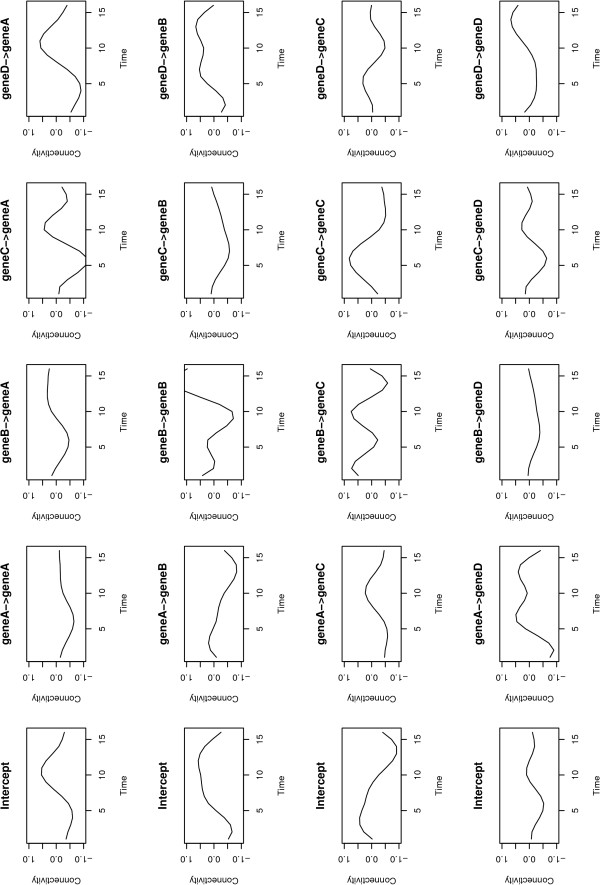
**DVAR output**. The output of DVAR showing the dynamics of the connectivity along time in four artificially generated genes where a connectivity above zero represents an induction and below zero represents repression.

• **Normalized microarray data: **The output is composed by graphical views of MA plots (raw data, normalized data and print-tips) and a file containing all the normalized expression data (Figure 2).

• **Differentially expressed genes: **Given the FDR-adjusted p-value threshold, GEDI provides an ordered list from the lowest to the highest level of significance (the most differentially expressed genes) adjusted by FDR [10].

• **Samples clustering and classification: **Statistics for each kind of analysis is provided, such as the number of corrected classified samples after cross-validation.

• **Gene expression regulatory networks: **GEDI plots graphs which represent the regulatory networks (Figure 3). Each node of the graph represents the gene, and the edges represent the Granger causalities (VAR, DVAR and SVAR) and correlations (Pearson and Spearman). It also plots the time-varying connectivity graphic, time × connectivity plot, to visualize how the connectivity changes with time in the DVAR method (Figure 4).

GEDI is very user-friendly, since all that is required is to upload GEDI in the R environment, leading it to automatically start running. Moreover, one may easily add new functionalities and extend GEDI.

As perspectives, we intend to continue the development of GEDI by incorporating new functionalities as soon as new algorithms and statistical methods are developed to analyze gene expression data, allowing and facilitating the access to advanced methods by biomedical researchers.

## Conclusion

GEDI was designed to be an integrated, user-friendly, viewer that combines the state of the art SVR, DVAR and SVAR algorithms. It facilitates the application of SVR, DVAR and SVAR, which used to be available only as cumbersome mathematical formulas, allowing to use them with visualizations for assessment of the results. Both running the statistical methods and visualizing of the results are accomplished within the graphical user interface, rendering the algorithms accessible to the large community of molecular biology researchers.

## Availability and Requirements

• Project name: GEDI

• Project homepage: 

• Operating systems: Windows and Linux

• Programming language: The software is implemented in R.

• Other requirements: The R environment and some R packages (tcltk2, splines, kernlab, sna, wavethresh, MASS, pvclust, samr, affy) which are freely available at .

• License: The license is distributed under the GNU General Public License.

• Any restrictions to use by non-academics: none

## Authors' contributions

AF has made substantial contributions to the conception, design and implementation of the study, and has also been responsible for drafting the manuscript. JRS has made substantial contributions to data analysis and implementation. CEF has discussed the results and critically revised the manuscript for important intellectual content and has given the final approval of the version to be published. MCS has directed the work and critically revised the manuscript for important intellectual content and has given the final approval of the version to be published. All authors read and approved the final manuscript.

## Supplementary Material

Additional file 1 This zipped file contains the GEDI R package. Click here for file

## References

[B1] Fujita A, Sato JR, Rodrigues LO, Ferreira CE, Sogayar MC (2006). Evaluating different methods of microarray data normalization. BMC Bioinformatics.

[B2] Fujita A, Sato JR, Garay-Malpartida HM, Morettin PA, Sogayar MC, Ferreira CE (2007). Time-varying modeling of gene expression regulatory networks using the wavelet dynamic vector autoregressive method. Bioinformatics.

[B3] Fujita A, Sato JR, Garay-Malpartida HM, Yamaguchi R, Miyano S, Sogayar MC, Ferreira CE (2007). Modeling gene expression regulatory networks with the sparse vector autoregressive model. BMC Systems Biology.

[B4] The R project for statistical computing. http://www.r-project.org/.

[B5] Bolstad BM, Irizarry RA, Astrand M, Speed TP (2003). A comparison of normalization methods for high density oligonucleotide array data based on variance and bias. Bioinformatics.

[B6] Yang YH, Dudoit S, Luu P, Lin DM, Peng V, Ngai J, Speed TP (2002). Normalization for cDNA microarray data: a robust composite method addresing single and multiple slide systematic variation. Nucleic Acids Res.

[B7] Baird D, Johnstone P, Wilson T (2004). Normalization of microarray data using a spatial mixed model analysis which includes splines. Bioinformatics.

[B8] Workman C, Jensen LJ, Jarmer H, Berka R, Gautier L, Nielser HB, Saxild HH, Nielsen C, Brunak S, Knudsen S (2002). A new non-linear normalization method for reducing variability in DNA microarray experiments. Genome Biology.

[B9] Wang J, Ma JZ, Li MD (2004). Normalization of cDNA microarray data using wavelet regressions. Combinatorial Chemistry & High Throughput Screening.

[B10] Benjamini Y, Hochberg Y (1995). Controlling the False Discovery Rate: A practical and powerful approach to multiple testing. J R Statist Soc B.

[B11] Tusher V, Tibshirani R, Chu G (2001). Significance analysis of microarrays applied to the ionizing radiation response. PNAS.

[B12] Fisher RA (1936). The use of multiple measurements in taxonomic problems. Annals of Eugenics.

[B13] Suzuki R, Shimodaira H (2004). An application of multiscale bootstrap resampling to hierarchical clustering of microarray data: How accurate are these clusters?. The Fifteenth International Conference on Genome Informatics.

[B14] Yeang CH, Ramaswamy S, Tamayo P, Mukherjee S, Rifkin RM, Angelo M, Reich M, Lander E, Mesirov J, Golub T (2001). Molecular classification of multiple tumor types. Bioinformatics.

[B15] Granger CWJ (1969). Investigating causal relation by econometric and cross-sectional method. Econometrica.

[B16] Mukhopadhyay ND, Chatterjee S (2007). Causality and pathway search in microarray time series experiment. Bioinformatics.

