# Supplementary material for: GEDI: a user-friendly toolbox for analysis of large-scale gene expression data
Source: BMC Bioinformatics. 2007 Nov 19;8:457. doi: 10.1186/1471-2105-8-457 (PMC2194737; doi:10.1186/1471-2105-8-457)
Supplement: Additional file 1 — This zipped file contains the GEDI R package. [file 1471-2105-8-457-S1.zip › gedi/inst/doc/GEDI_1.0_UserGuide.pdf]

GEDI 1.0 (Gene Expression Data Interpreter) User's Guide

You are welcome to use GEDI 1.0 to analyze your microarray data! If you have any questions, comments, suggestions or if you need help with GEDI, please contact us!

André Fujita	andrefujita@gmail.com
João Ricardo Sato	jrsatobr@gmail.com
Carlos Eduardo Ferreira	cef@ime.usp.br
Mari Cleide Sogayar	mcsoga@iq.usp.br

Terms of use

You may download and use GEDI for scientific and non-commercial purposes.
If you use GEDI in your research work, please cite appropriately.
GEDI is distributed under the GNU General Public License.

System requirements

To use GEDI, you must have the R program (version 2.6.0 or newer) installed in your computer. It is a freely available software (GPL), which may be downloaded at http://www.r-project.org. Choose the appropriate version for your operational system.

How to use GEDI

Firstly, download the GEDI package at http://www.iq.usp.br/wwwdocentes/mcsoga/gedi/

To run GEDI, first you will need to install and open R.

Download R from http://www.r-project.org and install.

Start the R program. On Windows, this usually means double-clicking on the R icon. On Linux, type "R" at a shell prompt.

After running R, install the following packages: affy, kernlab, MASS, pvclust, samr, sna, splines, tcltk2, wavethresh, otherwise, some of the procedures will not work. It may be installed easily using the installation script which we provide at GEDI homepage. This script will download and install all the required packages by GEDI.

To use this script, go to Menu and click on "Source code…".

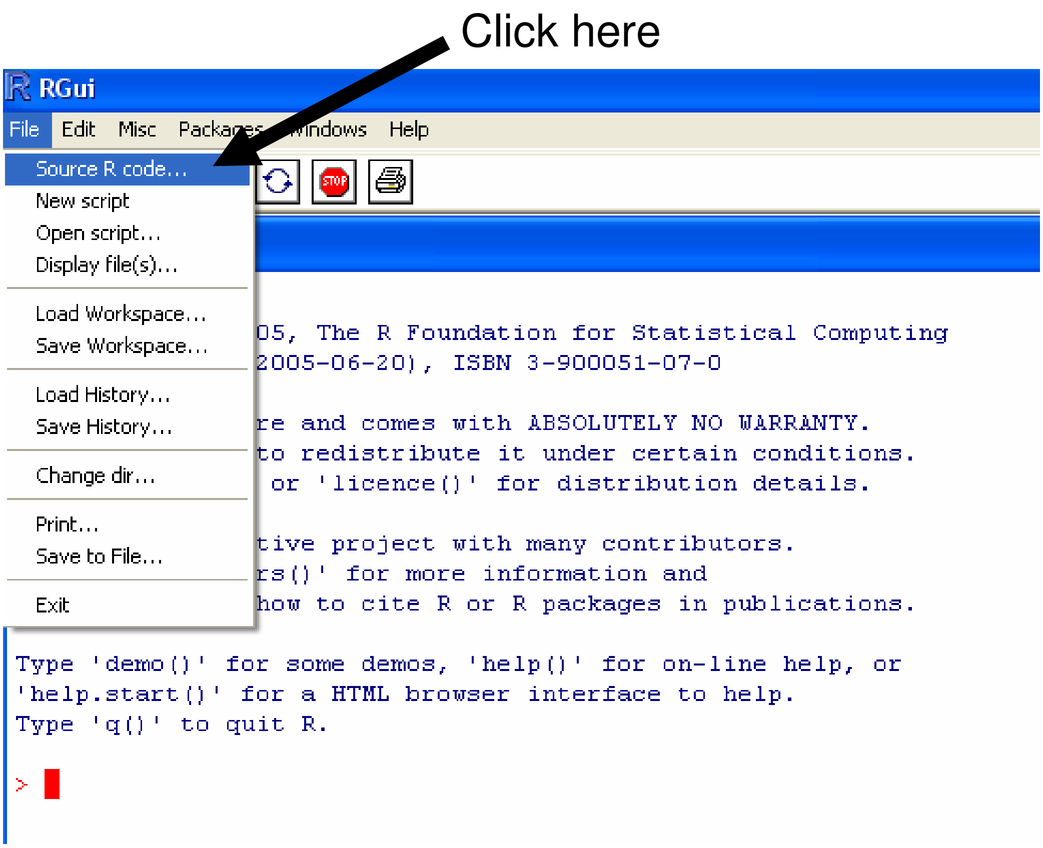

Figure 1: Installing the required packages.

Select the file "installPackages.R".

It will automatically start to download and install the packages.

If you use linux, just run the script "installPackages.R" in your R prompt.

To install GEDI (Windows), download the gedi.zip file at GEDI homepage. Then, go to the "Packages" menu and select "Install package(s) from local zip files…" and, finally, select the GEDI.zip.

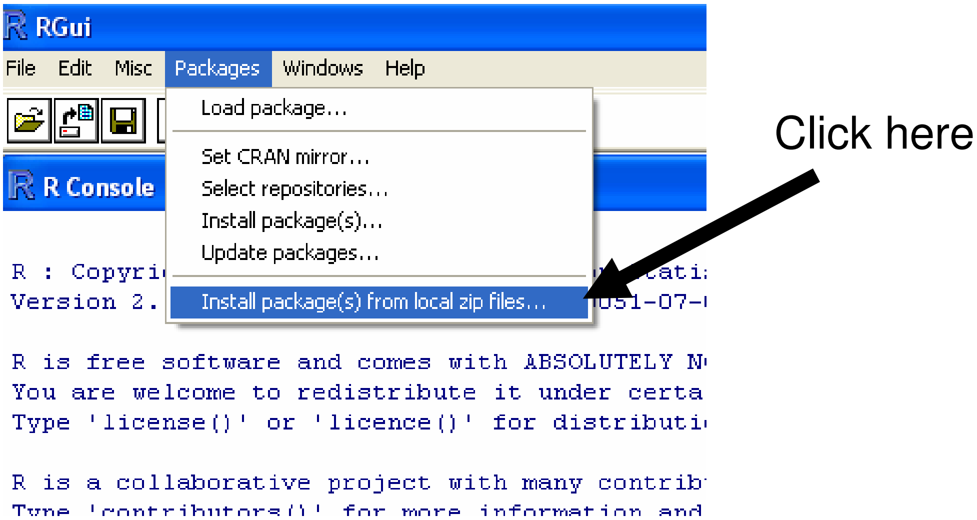

Figure 2: Installing GEDI package.

If you are using Linux, just type at your shell:
R CMD INSTALL GEDI.tar.gz

For both, Linux and Windows, call GEDI from the R terminal:
require(gedi)

Then, type:
gedi()

A graphical interface with an interactive menu will appear. This is GEDI!

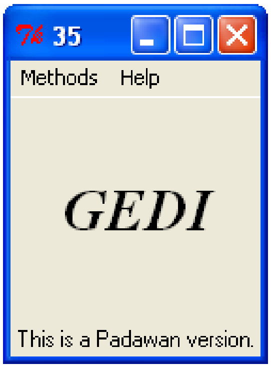

Figure 3: GEDI Gene Expression Data Interpreter graphical interface.

Data files format

Input and output files

The data files to be loaded MUST BE plain-text, tab or space delimited files. The first column MUST BE labeled with the **ID** for each gene. The first row MUST contain the microarrays labels. Missing values are not allowed. Decimals must be separated by dots.

The input file for normalization should not be log-transformed. Negative values are not allowed.

The input for differentially expressed genes and classification procedures are composed by TWO files. The first file must be the normalized data. The second one is a one column file. This column must contain the samples classes ("1" or "2").

Examples of input files may be found at the GEDI R package in the following directory: \gedi\data\Rdata.zip .

When the program asks the user to define an OUTPUT file, GEDI will create an XXX.ged file (where XXX is the file name provided by the user). This *.ged is a plain text, space delimited file and may be read by the common text editors such as Wordpad and or Excel spreadsheets. In addition to *.ged, GEDI

will create a *.log file containing the information about the procedure used and when the analysis was performed. If figures and/or graphics are provided, the user may save in a PostScript format which allows zooming without losing resolution.

For more information of input data format, see the examples provided with this distribution.

Data Visualization

The *.ged and *.log files may be visualized using common text editors or Excel spreadsheets, for instance. The *.ps files may be visualized using the GhostView software which is freely available at: http://pages.cs.wisc.edu/~ghost/.

Normalization

The graphical output will be automatically displayed when TWO microarrays are being normalized.

GEDI 1.0 provides 6 types of normalization: Loess, Splines, Wavelets, Support Vector Regression, Global (total intensity, median centering and mean centering) and Quantile procedures.

When performing normalization procedures, GEDI will always ask the user to define an output file. For normalization, the input file must be a TXT file. The first row should be the microarray labels and the first column should be the ID, i.e., repeated names are not allowed. For example, see normTest2.txt and normTestn.txt. Be sure that your file does not have missing and/or negative values and the decimals are separated by dots. If two microarrays will be normalized, MA plots are available. If more than two microarrays will be normalized, MA plots are not available and the cyclic normalization will be performed. This graph does not display interactive options. The graphical view is composed from left to right by: non-normalized MA plot, normalized MA plot, and print-tips. This graphical view could be saved in a *.ps file format. The output file contains the normalized microarray data in each column.

For details about these normalization procedures, see References:

Example files:
normTest2.txt file: to perform normalization of two microarrays.
normTestn.txt: to perform normalization of more than two microarrays.

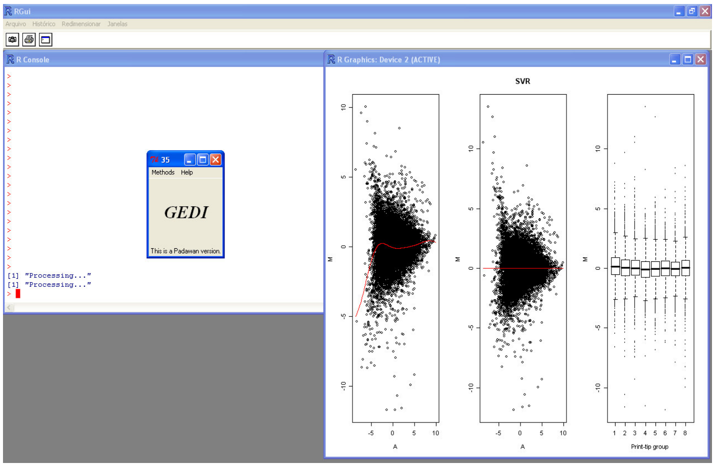

Figure 4: Result obtained running the SVR normalization method using normTest2.txt as input file.

References:

- Fujita et al. Evaluating different methods of microarray data normalization. BMC Bioinformatics. v7, 469.
- Bolstad B. Probe Level Quantile Normalization of High Density Oligonucleotide Array Data. <URL:http://oz.berkeley.edu/~bolstad/stuff/qnorm.pdf>
- Bolstad et al. Comparison of Normalization Methods for High Density Oligonucleotide Array Data Based on Bias and Variance. Bioinformatics. 19:185-193, 2003.

Inferring differentially expressed genes

GEDI will ask the q-value (FDR rate) threshold for t-test and Wilcoxon test. For SAM (Significance Analysis of Microarray), GEDI will ask for the delta value (for more details about delta, see Tusher *et al.*, 2001).

The input data must be two TXT files: one with gene expression data in columns. The first row should contain the arrays labels and the first column should contain the gene names. The other file should contain the samples classes in one column. Both files should have the samples in the same order. For example, see diffTest.txt and labelClass.txt. The output file contains the differentially expressed genes ordered from the lowest to the highest p-value (adjusted by FDR). Notice that only genes with q-value lower than the threshold set by the user is plotted.

t-test: performs a t-test with FDR (Benjamini and Hochberg, 1995) adjustment.
Wilcoxon test: performs a Wilcoxon test with FDR (Benjamini and Hochberg, 1995) adjustment.
t-test with permutation: performs a t-test with bootstrap approach and FDR adjustment (Benjamini and Hochberg, 1995).
SAM test: performs the SAM procedure (Tusher et al., 2001).

Example files:

diffTest.txt and labelClass.txt, where diffTest.txt contains gene expression and labelClass.txt contains t.

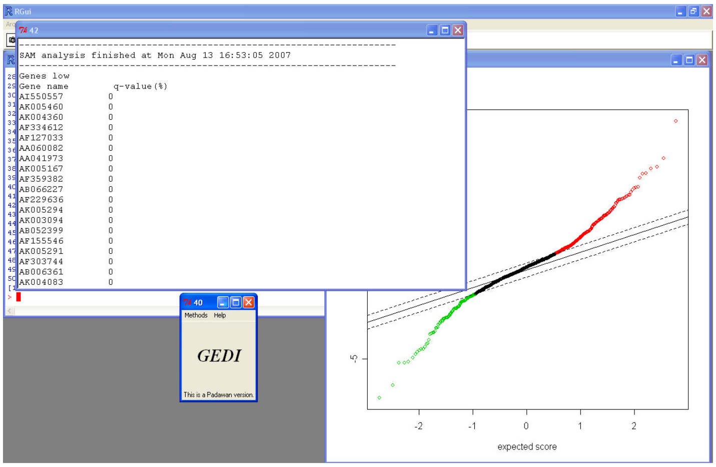

Figure 5: Result obtained running the SAM method using diffTest1.txt and diffTest2.txt as input files.

References:

- Benjamini Y, Hochberg Y. Controlling the false discovery rate: a practical and powerful approach to multiple testing. J. Roy. Statist. Soc. Ser. B. 57:289-300, 1995.
- Tusher et al. Significance analysis of microarrays applied to the ionizing radiation response. PNAS. 98:5116-5121, 2001.

Classifiers

GEDI is able to perform 3 types of samples classification, namely: k-means, linear/quadratic Fisher discriminant analysis and Support Vector Machine (SVM). A cross-validation procedure is performed to quantify the accuracy of the predictors.

The input data must be two TXT files: one with gene expression data in columns. The first row should contain the arrays labels and the first column should contain the gene names. The other file should contain the samples classes in one column. Both files should have the samples in the same order. For example, see classTest.txt and labelClass.txt. The output file contains the samples names, the "true" classification and the predicted classification by cross validation. Missing values are not allowed. For example of an input file, see classTest.txt.

K-means: performs a k-means clustering.

Fisher discriminant analysis: performs linear and/or quadratic Fisher discriminant analysis (Fisher, 1936).

SVM: performs the Support Vector Machine analysis (Brown et al., 2000).

Example files:

classTest.txt, labelClass.txt

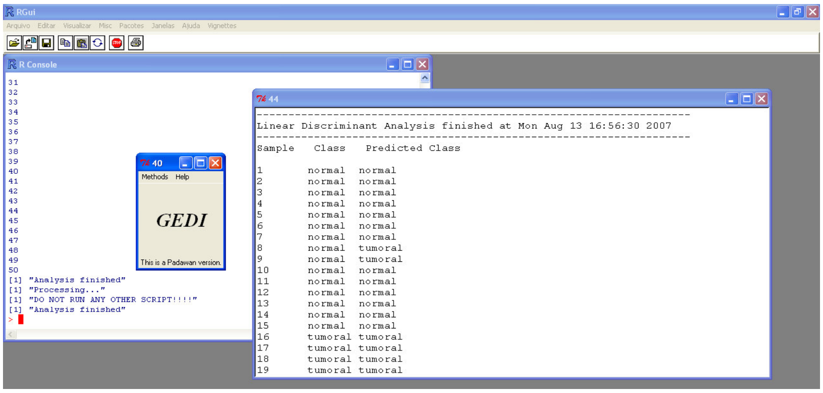

Figure 6: Result obtained running Fisher discriminant analysis method using classTest.txt as input file.

<u>References:</u>

- Brown et al. Knowledge-based analysis of microarray gene expression data by using support vector machines. Proc. Natl. Acad. Sci. 97:262-267, 2000.
- Fisher RA. The use of multiple measurements in taxonomic problems. Annals of Eugenics. 7:179-188, 1936.

Clustering analysis

Hierarchical clustering based on multi-scale bootstrap re-sampling is provided. For more details about this procedure, see: Shimodaira et al., (2002, 2004) and Suzuki & Shimodaira, 2004.

For the user, a dendogram is plotted in addition to numerical results, which may be read in common text editors.

The input file should be a TXT file. The first row should contain the microarrays labels, and the first column should contain the gene names. Missing values are not allowed. For example of an input file, see clustTest.txt.

Hierarchical: calculates p-values for hierarchical clustering via multiscale bootstrap resampling.

Example file:
clustTest.txt

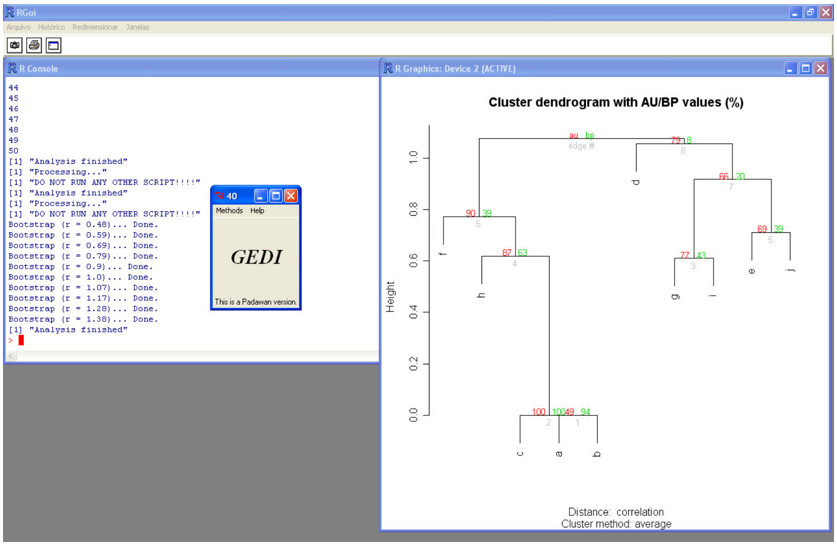

Figure 7: Result obtained running the Hierarchical clustering method using clustTest.txt as the input file.

<u>References:</u>

- Shimodaira H. Approximately unbiased tests of regions using multistep multiscale bootstrap resampling. Annals of Statistics, 32:2616-2641, 2004.
- Shimodaira H. An approximately unbiased test of phylogenetic tree selection. Systematic Biology, 51:492-508, 2002.
- Suzuki R and Shimodaira H. An application of multiscale bootstrap resampling to hierarchical clustering of microarray data: How accurate are these clusters? The Fifteenth International Conference on Genome Informatics 2004. P034, 2004.

Connectivity models

To infer gene expression regulatory networks, GEDI 1.0 provides 3 methods based on time series data and 4 methods which may or may not be a time series data, namely VAR (Vector Autoregressive model), DVAR (Dynamic Vector Autoregressive model), SVAR (Sparse Vector Autoregressive model), Partial Pearson and Spearman correlation and pairwise Pearson and Spearman correlation analysis. For VAR, DVAR and SVAR, each column of the input data must be a time point of the time series, displayed in an increasing order from the left column to the right column in the matrix. The time series must be equally spaced.

The input file should be a TXT file with the microarray data in columns with no missing values and ordered by the time series. For example, see netTest.txt.

The ouput file is a file containing the adjacency matrix of significant connectivities (given the q or p

value threshold). It is important to notice that for VAR, DVAR and SVAR, the samples should be a time-series data, whereas for correlation analysis, they may or may not be a time series data.

The p-value matrix represents the connectivity between row *i* (gene) and column *j* (gene).
In the p-value matrix of DVAR, the first column represents the p-values for the intercept. The following two columns represent the p-values of the first gene. The following two columns correspond to the second gene and so on. Notice that only p-values higher than 0 and lower than the p-value threshold set by the user are plotted. Other values are printed as 0.

VAR: the Granger Causality routine estimates a Vector Autoregressive model of order 1.
DVAR: estimates the network by Dynamic Vector Autoregressive method of order 1 (Fujita et al., 2007a).
SVAR: estimates the network by Sparse Vector Autoregressive method of order 1 (Fujita et al., 2007b).
Partial Correlations: Calculate Spearman's and Pearson's Partial Correlations between the columns of the input file.
Single Correlations: Calculate Spearman's and Pearson's Correlation between the columns of the input file.

Example files:
netTest1.txt

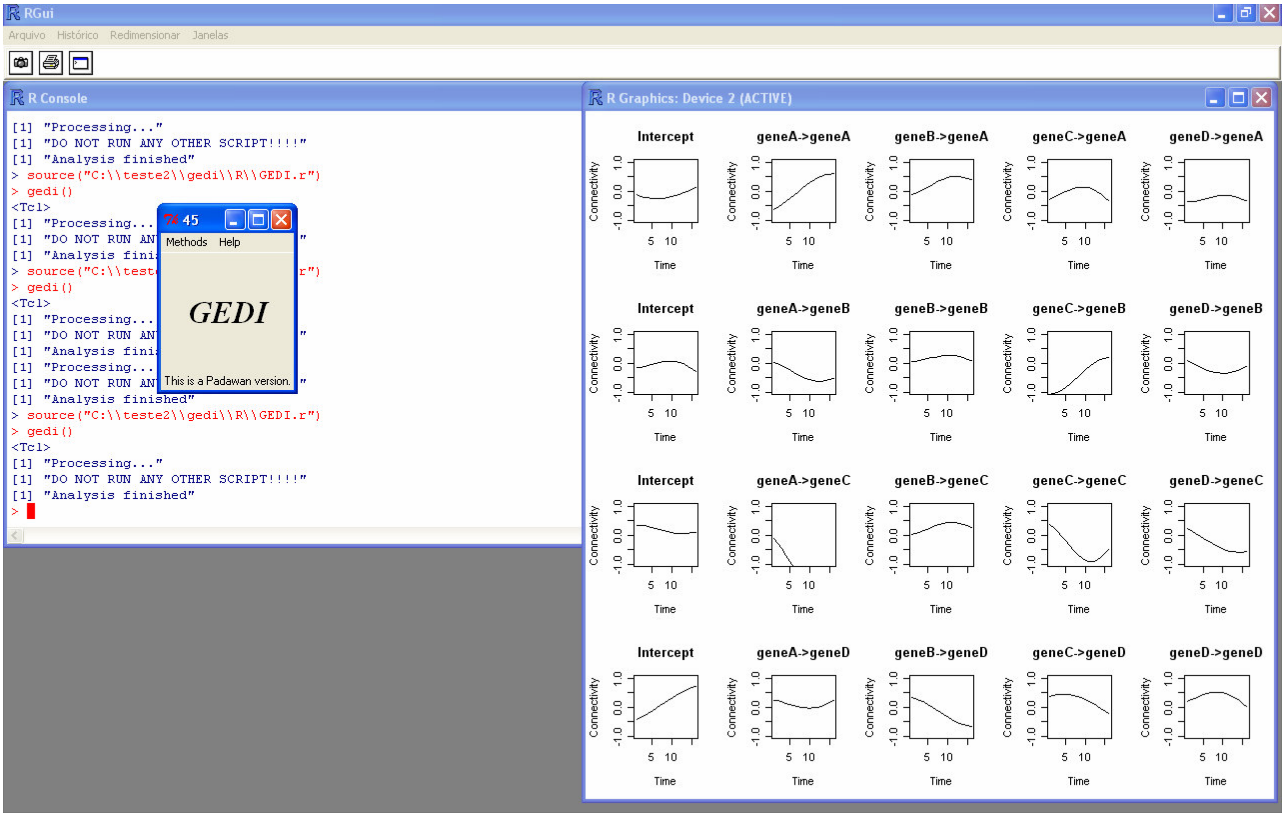

Figure 8: Result obtained running DVAR method using netTest1.txt as input file.

Table 1: Coefficient and p-value matrix obtained running pearson partial correlation with netTest2.txt. These matrix could be interpreted as adjacency matrix where there is a linkage from row *i* (gene *i*) to column *j* (gene *j*).

Coefficient_matrix

1	0.025717	0.017358	0.601072
0.025717	1	0.560696	-0.15896
0.017358	0.560696	1	0.08586
0.601072	-0.15896	0.08586	1

p-value_matrix

0	0.86683	0.909894	1.27E-05
0.86683	0	6.18E-05	0.296973
0.909894	6.18E-05	0	0.574936
1.27E-05	0.296973	0.574936	0

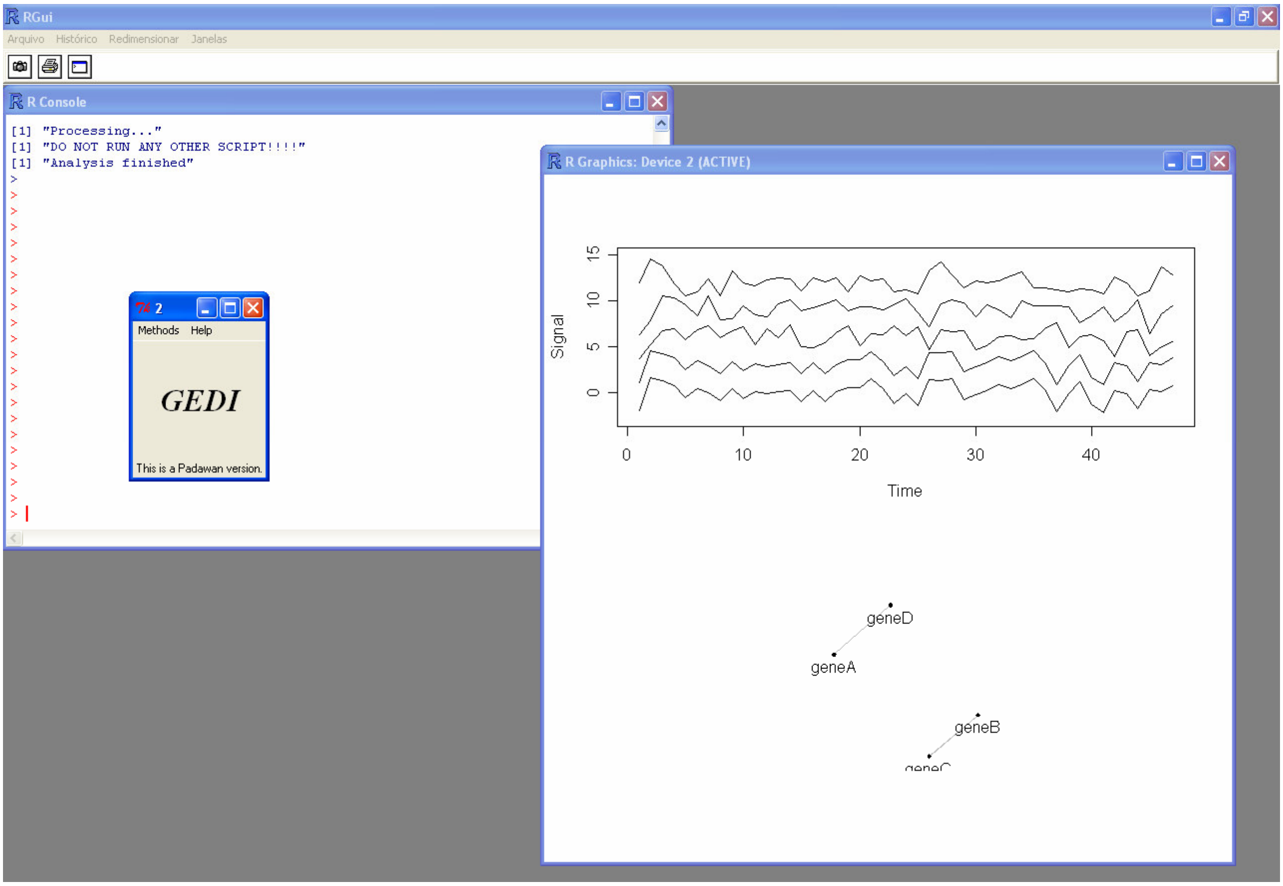

Figure 9: Result obtained running pearson partial correlation method using netTest2.txt as input file.

References:

- Fujita et al. Time-varying modeling of gene expression regulatory networks using the wavelet dynamic vector autoregressive method. Bioinformatics. 23:1623-1630, 2007a.
- Fujita et al. Modeling gene expression regulatory networks with the sparse vector autoregressive model. BMC Systems Biology, 1:39, 2007b.

Help – How to

To access Help functionalities, click on the "Help" menu, then choose the "How to" menu and select the item of interest. Then, a window containing instructions related to the selected item will pop up.

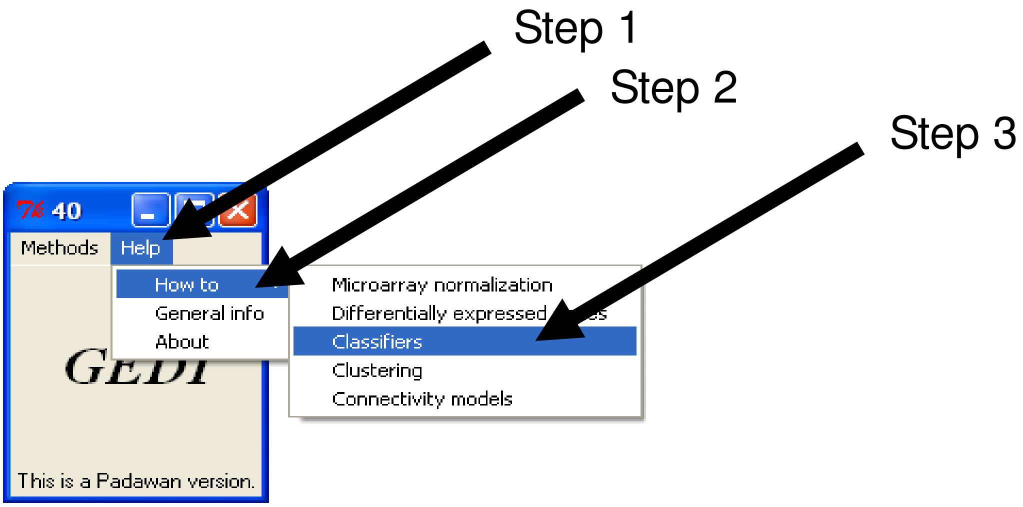

Figure 10: To access Help functions.

Frequently Asked Questions (Troubleshooting)

1. **GEDI is not loading the input file.**

Verify the input file format. The first row must contain the genes/probes IDs. Repetitive ID's are not allowed. Then, verify if the first row contains the microarrays labels. Repetitive labels are not allowed. Then, verify if the IDs or labels contain "spaces". "Spaces" are not allowed. Set "microarray_1" instead of "microarray 1", for instance. Missing values are not allowed either.
Finally, certify that the decimals are separated by dots and the data is tab/space delimited.

2. **GEDI prints error messages such as "could not find function XXX".**

Verify if ALL the packages required by GEDI (see **System requirements** section) are properly installed in your computer before running GEDI.

3. **Normalization methods are not plotting the MA plots.**

Remember that MA graphics are plotted only when TWO microarrays are normalized. For more than two microarrays, only the numerical results are printed in a file defined by the user.

4. **I do not know whether GEDI is running. Nothing happens.**

Depending on your machine configuration, some functionalities may require a considerable time (SVR normalization, hierarchical clustering, differential expression inference procedures depending on the number of genes). GEDI plots the message "Processing" in your R terminal when the analysis starts and plots a message "Analysis finished" in a pop-up window and in the R terminal to alert you that it was concluded. Be patient and do not run any other script in the R environment while the GEDI procedure is running. We recommend to running the procedures overnight if you are interested in analyzing a large amount of data.

R functions {packages} used in GEDI

bs {splines}: not informed.
gplot {sna}: developed by Butts CT and Montgomery A.
ksvm {kernlab}: developed by Karatzoglou A.
loess {stats}: developed by Ripley BD.
normalize.quantiles {affy}: developed by Bolstad B.
pvclust {pvclust}: developed by Suzuki R.
samr {samr}: developed by Narasimhan B and Tibshirani R.
wd {wavethresh}: developed by Nason G..

References

- Bolstad B. Probe Level Quantile Normalization of High Density Oligonucleotide Array Data. <URL:http://oz.berkeley.edu/~bolstad/stuff/qnorm.pdf>
- Bolstad *et al*. Comparison of Normalization Methods for High Density Oligonucleotide Array Data Based on Bias and Variance.Bioinformatics 19:185-193 2003.
- Brown *et al*. Knowledge-based analysis of microarray gene expression data by using support vector machines. Proc. Natl. Acad. Sci. 97:262-267, 2000.
- Fujita *et al*.Evaluating different methods of microarray data normalization.BMC Bioinformatics. v7, 469.
- Fujita *et al*. Time-varying modeling of gene expression regulatory networks using the wavelet dynamic vector autoregressive method. Bioinformatics. 23:1623-1630, 2007.
- Fujita *et al*. Modeling gene expression regulatory networks with the sparse vector autoregressive model. BMC Systems Biology, 1:39, 2007.
- Shimodaira H. Approximately unbiased tests of regions using multistep-multiscale bootstrap resampling. Annals of Statistics, 32:2616-2641, 2004.
- Shimodaira H. An approximately unbiased test of phylogenetic tree selection. Systematic Biology, 51:492-508, 2002.
- Suzuki R and Shimodaira H. An application of multiscale bootstrap resampling to hierarchical clustering of microarray data: How accurate are these clusters? The Fifteenth International Conference on Genome Informatics 2004. P034, 2004.
- Tusher *et al*. Significance analysis of microarrays applied the ionizing radiation response. PNAS. 98:5116-5121, 2001.
